# Host genetic variation and its microbiome interactions within the Human Microbiome Project

**DOI:** 10.1186/s13073-018-0515-8

**Published:** 2018-01-29

**Authors:** Raivo Kolde, Eric A. Franzosa, Gholamali Rahnavard, Andrew Brantley Hall, Hera Vlamakis, Christine Stevens, Mark J. Daly, Ramnik J. Xavier, Curtis Huttenhower

**Affiliations:** 1 Center for Computational and Integrative Biology, Massachusetts General Hospital, 185 Cambridge St, Boston, MA 02114 USA; 2 Department of Biostatistics, Harvard T. H. Chan School of Public Health, 655 Huntington Ave, Boston, MA 02115 USA; 3 The Broad Institute of MIT and Harvard, 415 Main St, Cambridge, MA 02142 USA; 4 Center for Human Genetic Research, Massachusetts General Hospital, 185 Cambridge St, Boston, MA 02114 USA; 5 Center for Microbiome Informatics & Therapeutics, Massachusetts Institute of Technology, Cambridge, MA 02139 USA

**Keywords:** Human Microbiome Project, Microbiome and human genetics, Human genome sequence, Microbiome metagenome sequence, Association studies

## Abstract

**Background:**

Despite the increasing recognition that microbial communities within the human body are linked to health, we have an incomplete understanding of the environmental and molecular interactions that shape the composition of these communities. Although host genetic factors play a role in these interactions, these factors have remained relatively unexplored given the requirement for large population-based cohorts in which both genotyping and microbiome characterization have been performed.

**Methods:**

We performed whole-genome sequencing of 298 donors from the Human Microbiome Project (HMP) healthy cohort study to accompany existing deep characterization of their microbiomes at various body sites. This analysis yielded an average sequencing depth of 32x, with which we identified 27 million (M) single nucleotide variants and 2.3 M insertions-deletions.

**Results:**

Taxonomic composition and functional potential of the microbiome covaried significantly with genetic principal components in the gastrointestinal tract and oral communities, but not in the nares or vaginal microbiota. Example associations included validation of known associations between FUT2 secretor status, as well as a variant conferring hypolactasia near the *LCT* gene, with *Bifidobacterium longum* abundance in stool. The associations of microbial features with both high-level genetic attributes and single variants were specific to particular body sites, highlighting the opportunity to find unique genetic mechanisms controlling microbiome properties in the microbial communities from multiple body sites.

**Conclusions:**

This study adds deep sequencing of host genomes to the body-wide microbiome sequences already extant from the HMP healthy cohort, creating a unique, versatile, and well-controlled reference for future studies seeking to identify host genetic modulators of the microbiome.

**Electronic supplementary material:**

The online version of this article (10.1186/s13073-018-0515-8) contains supplementary material, which is available to authorized users.

## Background

The Human Microbiome Project (HMP) was the first population-scale, body-wide metagenomic microbiome survey, with initial results published in 2012 [1]. Covering 18 clinically relevant body sites from five major body regions in a cohort of 300 healthy adult donors, analysis of HMP data revealed a high degree of microbial community specialization as well as considerable variation in overall microbiome composition between individuals. Providing a baseline of healthy microbial variation, HMP data have served as a versatile reference in numerous studies [2–5]. Examples of how metagenomic data from the HMP have been used include characterizing mobile gene content in the microbiome [6], identifying the prevalence of specific enzymes in human microbiome samples [7, 8], dissecting factors that shape skin microbial communities [9], contrasting ancient and modern oral microbiomes [10], and studying the gut antibiotic resistome [11].

In this study, we provide further data to characterize potential interactions between the microbiome and the human host, specifically, genome sequencing for the HMP cohort. While interpersonal variation in the microbiome can be considerable, the microbiome of specific individuals can conversely be remarkably stable [12–14], suggesting host genetic background as one factor maintaining the composition of microbial communities across the body. Genetic factors influencing microbiome composition have been previously analyzed in mouse models, where external factors such as diet can be tightly controlled. In these studies, host genotype explained a significant proportion of variation in the gut microbiome of intercrossed mouse lines [15–17]. The quantitative trait loci (QTLs) emerging from these studies included genes involved in immune function, for example, *Irak3* [15] and *Irak4* [17].

The study of genetic effects on the microbiome becomes far more complex in humans. In addition to genetics, microbiome composition is strongly influenced by environmental factors such as diet, overall health status, and medication use [18, 19]. With the exception of research on special populations like the Hutterites [20], these factors are difficult to constrain in a human study. Nevertheless, some evidence does exist for genetic effects on human microbiome composition. For example, the gut microbiomes of monozygotic twins are significantly more similar than those of dizygotic twins [21, 22]. Recent work has suggested that microbiome heritability is lower than many other traits, however, and not distributed equally among taxa (e.g., with a higher heritability among Firmicutes than Bacteroidetes [22, 23]).

More detailed mapping of associations between single gene variants and microbial taxa has been successful in a targeted approach of candidate genes or variants, where clear molecular mechanisms have been established. For example, inflammatory bowel disease risk loci near *NOD2* were also associated with *Enterobacteriaceae* in patients with inflammatory bowel disease [24]. In addition, variants near the lactase (*LCT*) gene, responsible for lactose tolerance, have been associated with abundance of *Bifidobacterium* [22, 25, 26]. *Bifidobacterium* has also been associated with a loss-of-function variant in the fucosyltransferase 2 (*FUT2*) gene, responsible for the transfer of the terminal fucose residues to the mucosal glycans [27].

Despite these examples, very few individual microbe-polymorphism associations have been identified that have reached genome-wide significance. Cohort size is certainly a main limiting factor. Studies to date have included up to 1800 individuals, a relatively small number for a successful genome-wide association study of any trait, and especially so considering that the heritability of microbial features can be low relative to many quantitative traits. Furthermore, clinically relevant associations might be discoverable only in cohorts with particular diseases, for example, in conditions such as inflammatory bowel disease or rheumatoid arthritis, which are both genetically complex and accompanied by microbial dysbioses [28, 29].

To facilitate future microbiome-genetic association meta-analyses and to provide a baseline characterization of the HMP population, we report here on whole-genome sequencing from the blood of 298 HMP participants. The data achieve an average of 32x coverage, allowing us to discover two times as many variants as were previously identified using “contaminant” human reads from a subset of 93 whole metagenome sequencing (WMS) samples [25, 30]. The common variants we identified are consistent with findings from other large-scale sequencing projects such as 1000 Genomes [31] and Genome of the Netherlands (GoNL) [32]; in addition, we identified numerous novel rare variants in the HMP cohort. In combination with more than 7500 microbiome samples sequenced (including both 16S rRNA gene sequencing and WMS) from multiple body sites of the HMP cohort to date, this study creates a unique dataset for studying the microbiome of multiple body sites in the context of host genetics.

## Methods

### Genome sequencing

Genome sequencing was performed at the Broad Institute using polymerase chain reaction (PCR)-free library preparation on Illumina HiSeq X Ten machines. Reads were mapped to the genome using Burrows-Wheeler Aligner [33], and variants were called with Genome Analysis Toolkit (GATK) version 3.4 on human genome build b37. The sequencing quality was good in all samples according to multiple metrics, so no samples were excluded. We used only variants that passed Variant Quality Score Recalibration filtering. Additionally, we filtered out variants in low-complexity regions of the genome as defined by Li et al. [34]. For comparison analyses, we downloaded variant files from the GoNL consortium (https://molgenis26.target.rug.nl/downloads/gonl_public/variants/release5/) and 1000 Genomes phase 3 (ftp://ftp.1000genomes.ebi.ac.uk/vol1/ftp/release/20130502/) and searched for the presence of all our variants in these other cohorts.

We estimated the impact of coding variants using Variant Effect Predictor (VEP) [35] with Ensembl version 82 together with the Loss-Of-Function Transcript Effect Estimator (LOFTEE) plugin (https://github.com/konradjk/loftee). To classify mutations into severity groups, we used the annotations provided by VEP. If one variant was located within several transcripts and therefore had multiple potential effects on the coding sequence, we annotated the variant with the most severe outcome.

For principal component analysis (PCA) on the variants, we used 5.9 million (M) common (minor allele frequency (MAF) > 0.05, call rate > 95%) single nucleotide variants (SNVs) in PLINK 1.9 [36]. For joint PCA, we first extracted a similar subset of 6.8 M SNVs from the 1000 Genomes data (MAF > 0.05, call rate > 95%), merged the resulting files, and performed PCA using PLINK 1.9.

Kinship analysis was performed using the Kinship-based INference for Genome-wide association studies (KING) algorithm [37]. The ranges used for inferring degree of relation from kinship coefficients were taken from the original publication and were [0.0442, 0.0884) for third degree, [0.0884, 0.177) for second degree, and [0.177, 0.354) for first degree relatives, and > 0.354 for twins. Kinship coefficients below 0.0442 were considered unrelated.

### Microbiome data

All metagenomic data underwent quality control according to the HMP protocol [38, 39]. We performed taxonomic profiling of bacteria, archaea, microbial eukaryotes, and viruses using MetaPhlAn2 [40]. Briefly, MetaPhlAn2 maps shotgun metagenomic sequencing reads against a precomputed database of clade-specific marker genes (i.e., genes that tend to be found in isolate genomes from a given clade, but are rarely seen in isolate genomes outside that clade). Marker gene abundance is averaged within-clade to produce a robust estimate of the clade’s genomic coverage in the sample, which can then be normalized to relative abundance units.

We used MetaPhlAn2’s taxonomic profiles to guide species-resolved functional profiling with HMP Unified Metabolic Analysis Network 2 (HUMAnN2) (http://huttenhower.sph.harvard.edu/humann2). For a given metagenome, HUMAnN2 constructs a sample-specific database by concatenating and indexing the pangenomes of species detected in the sample (species’ pangenomes are precomputed, reduced representations of the protein-coding sequences from isolates of a given species [41]). HUMAnN2 then maps sample reads against the corresponding sample-specific database to quantify gene presence and abundance on a per-species basis; reads that fail to map to one of the detected species (“unclassified reads”) are separately mapped by translated search against a reduced protein sequence catalog [42]. Finally, HUMAnN2 compares community total, species-resolved, and unclassified gene family abundance to the MetaCyc pathway catalog [43] to reconstruct metabolic pathway abundance and coverage using the original HUMAnN algorithm [44].

To filter the MetaPhlAn relative abundances for further testing, we used only species-level data and required a species to be present in at least 25% of the samples in a given body site. This reduced the number of species from 567 to 119 in stool, from 428 to 119 in buccal mucosa, from 479 to 161 in supragingival plaque, from 461 to 156 in tongue dorsum, from 380 to 29 in anterior nares, and from 367 to 23 in posterior fornix. We then applied log_10_ transformation on their relative abundances (with a pseudocount of 10^–5^) to stabilize the variation for linear modeling.

Metabolic pathway abundance was much more stable than species-level abundance: for example, more than 78% of the pathways detected were present in more than 75% of stool samples. As the number of samples where a pathway was not detected was relatively small for each pathway, we used only the abundances that were present for statistical testing, and used only pathways that were present in more than 75% of the samples per body site. This reduced the number of species from 756 to 500 in stool, from 706 to 427 in buccal mucosa, from 750 to 511 in supragingival plaque, from 744 to 509 in tongue dorsum, from 742 to 355 in anterior nares, and from 697 to 247 in posterior fornix. Finally, we applied log_10_ transformation to the read-counts-per-million pathway abundance values returned by the HUMAnN2 pipeline.

In both cases, if multiple samples were collected from the same person and body site, we averaged the relative abundance values. The final number of donors was 209 for stool, 159 for buccal mucosa, 169 for supragingival plaque, 185 for tongue dorsum, 128 for anterior nares, and 80 for posterior fornix.

### Genome and microbiome associations

To associate genomic PCA with the microbiome, we fit a linear model to each of the microbial features, predicting it using the first five principal components. Based on the residuals, we calculated the amount of variance explained (*R*^2^). To put the calculated values into context, we shuffled the sample labels 10,000 times and calculated *R*^2^ for each of the shuffled datasets. The average *R*^2^ statistic per site was averaged over all features, and the Z-score was calculated based on the same statistic in the 10,000 permuted datasets. For single features the empirical *p* value was calculated as the proportion of permuted *R*^2^ scores larger than the actual score.

Starting with ranking of pathways based on their correlation to genomic principal components, we sought to identify classes enriched in the top of the rankings. For this analysis we used the pathway superclass assignments by MetaCyc [43]. For each superclass in the data, we extracted the rankings of its members and calculated a *p* value [45] showing how strongly the rankings were skewed towards the top.

For genome-wise associations we employed Matrix eQTL software [46] to fit ordinary linear models to predict microbiome features, taking originating site, sex, and ethnicity into account as covariates. Given the set of donors available for each body site, we included only the SNVs with MAF > 0.1. The variants found in the analysis were associated with genes that were located within 50 kb of the variant. The variants reported in figures and supplementary tables were filtered as follows: (1) we divided all SNVs associated to a particular species into groups where the distance between consecutive SNVs was not longer than 10,000 bp; (2) from each group of associations, we selected the one with the smallest *p* value.

## Results

### High-quality DNA sequencing of the HMP cohort

The HMP cohort design and sample collection has been described in depth [47]. Briefly, the HMP cohort comprises 300 donors recruited in two locations in the USA. The majority of donors (71%) were of Caucasian origin; the remaining donors were of African (6%), Asian (9%), Latino (11%), or mixed (3%) ancestry. The male-to-female ratio was roughly equal, with 151 females and 149 males. The goal in selecting donors was to find healthy individuals with no recent medication use or disease history, who belonged to a similar age group (19–40 years), and who had a relatively healthy body mass index (BMI of 19–34 kg/m^2^).

**Fig. 1:**
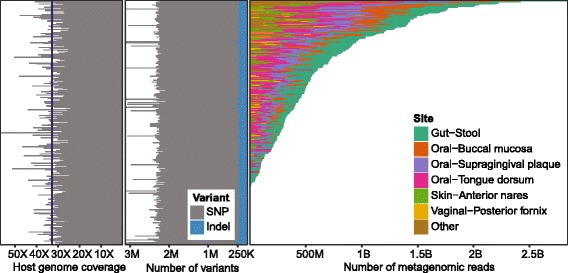
Overview of the Human Microbiome Project host genome and metagenome coverage. Sequencing depth for each host genome (*left*) and number of reads for all available samples with whole metagenome sequencing

To obtain host genome information, genomic DNA from the blood of 298 of the 300 individuals was sequenced using PCR-free sequencing. The average sequencing coverage was 32.77x, with a range of 23.9× to 56.7× (Fig. 1). Contamination and the percentage of chimeric reads were both well under the standard cutoff of 5% in all samples (Additional file 1: Figure S1A). The distribution of other quality metrics such as insert size and percentage of reads that aligned in pairs did not highlight any clear outlier samples; therefore, all samples were included in further analysis. The variant number was also remarkably stable at ~ 2 M single nucleotide polymorphisms (SNPs) and 200 K indels per person (Fig. 1), with the exception of the African-American donors, who had higher genetic diversity (Additional file 1: Figure S1B). There was no detectable correlation between sequencing depth and number of variants recovered, indicating that depth in all samples was sufficient.

These data provide an almost complete pairing of human genome sequencing to microbial amplicons and metagenomes across the entire HMP cohort. Genetic variation in this cohort was previously inferred using “contaminant” human reads from 93 subjects’ WMS data [25, 30]. While this provided an average human genome coverage of ~ 10×, it varied greatly between samples and for many reached only 5×. This was sufficient to detect 13 M genetic variants overall, 5.5 M with MAF > 0.05. In comparison, our study more than tripled the number of donors, and by directly targeting the host DNA, we identified twice as many variants overall with even coverage (minimum 25×) between samples. This increased the quality over all samples and generated a complete dataset that can be mined in this work as well as in future studies.

### Sequencing results are consistent with those of other comparable populations

**Fig. 2:**
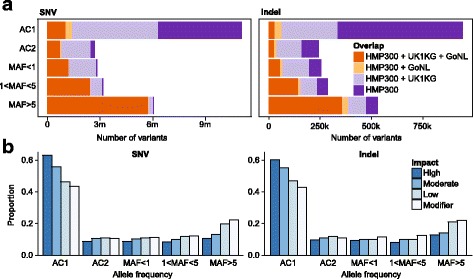
Distribution of genetic variants and comparison with other cohorts. **a** Discovered variants categorized by frequency and overlap with other cohorts. *AC* allele count, *MAF* minor allele frequency. **b** Distribution of the number of coding mutations by frequency and estimated impact

After filtering according to quality and location in low-complexity regions, 29 M variants remained, consisting of 26.7 M SNVs and 2.3 M insertions-deletions (indels) (Additional file 1: Table S1). When compared to the GoNL [32] and the 1000 Genomes [31] Projects, 5.1 M SNVs and 856 K indels were novel, but the majority of these were rare (Fig. 2a). In contrast, the common variants we identified (MAF > 5%) were almost universally shared between the three cohorts. Overall, we identified 7.8 M more variants compared to the similarly sized GoNL consortium. Since many of the variants were also present in the 1000 Genomes Project, we attribute the difference to the greater ethnic diversity in the HMP cohort. Although a large number of SNVs were unique to each cohort, the proportion of variants falling in intronic, exonic, and intergenic regions of the genome was almost identical between cohorts (Additional file 1: Figure S2).

We next annotated coding variants using the LOFTEE plugin for the VEP tool [35], which categorizes variants into classes based on their impact on the coding sequence. The number of high-impact variants, defined as those that would result in loss of function of a particular gene, was 2670 (Additional file 1: Table S2); this result is consistent with active negative selection against these variants. Negative selection was also evident from the allele frequency distribution, as the severity of an allele’s impact was strongly related to its frequency in the population. For example, high-impact variants were greatly enriched in variants that were observed only once in our dataset (Fig. 2b, AC1). The distribution of coding mutations among genes was also not uniform, with a small number of genes capturing a large number of variants. Thirty genes showed more than five potential high-impact loss-of-function variants, and six genes had more than ten variants. The small number of genes with high-impact coding mutations suggested that this cohort was too small for burden testing to draw correlations between mutation frequency within a gene and microbial features. Instead, we focused our analysis on identifying associations between common variants and microbial taxa or functional potential.

### Microbial taxa and functional potential at six body sites

For the HMP, microbiome samples were collected from 18 body sites, falling into five major areas: gastrointestinal (GI) tract, oral cavity, skin, nares, and vagina. In some cases replicate samples were collected over time to assess temporal stability of the microbiome. In total, more than 5000 samples were characterized using 16S rRNA gene sequencing and more than 2000 using shotgun WMS. The former approach gives a high-level overview of taxonomic composition, whereas the latter allows species-level identification and profiling of functional potential of the microbiome. We therefore used WMS data in subsequent analyses. The distribution of samples with WMS was not equivalent between body sites, with most samples drawn from six locations representing four of the major areas described above: gut (stool), oral (buccal mucosa, supragingival plaque, tongue dorsum), nares (anterior nares), and vaginal (posterior fornix); no WMS data were available from the skin samples. Within the six body sites, the number of donors ranged from 80 for vaginal posterior fornix to 209 for gut samples; the average number of reads per sample ranged from 34 M in posterior fornix to 86 M in tongue dorsum. Using the WMS data from these samples, we identified taxonomic composition using MetaPhlan2 [40] and functional potential using HUMAnN2 [44]. These outputs were then analyzed for associations with host genetic variation.

### Human genomic principal components correlate with microbiome composition

**Fig. 3:**
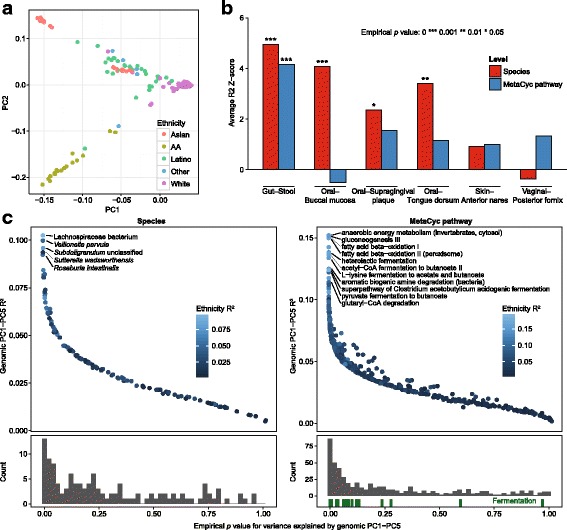
Correlation between high-level genetic variation and microbiome composition. **a** The first two components of the genetic principal component analysis are shown, based on common single nucleotide variants, overlaid by self-reported donor ethnicity. *AA* African-American. **b** Shown is how much variance in microbiome data on average can be explained by the genetic principal components, when compared to permutation on the same data. Values shown are Z-scores based on permutations, which were also used to calculate empirical *p* values. **c** Distribution of genetic principal component *R*^2^ values for different species and pathways in stool. *Y*-axis shows the variance explained, and the *X*-axis shows permutation-based empirical *p* values for each of those numbers. Only the names of species with false discovery rate (FDR) < 0.05 and pathways’ FDR < 0.01 are shown. The histogram *below* displays the distribution of empirical *p* values, and the *Y*-axis shows the number of species in a bin. *Green bars* under the pathway histogram show how the pathways that are associated with fermentation are ranked by *R*^2^

To compare host genetic variation with microbial variation, we first assessed the degree to which high-level genetic patterns could be correlated with microbiome composition. PCA on the common SNVs (MAF > 0.05) demonstrated that the first five principal components predominantly represented the ethnic and racial ancestry of the donors. For example, host genetics of the African-American, one of two groups of Asian-American, and the Caucasian subjects showed the strongest effect (Fig. 3a). To further compare overall genetic variation to other cohorts, we also jointly ordinated a combined dataset of HMP300 and 1000 Genomes participants, using SNVs with MAF > 0.05 in both cohorts. Individuals from both cohorts distributed in the resulting principal component space almost identically according to ancestry (Additional file 1: Figure S3).

Next, for HMP300 we calculated what percentage of microbial variation in the six body sites could be explained (*R*^2^) by the first five host-genome principal components. In stool samples, the percent of species-level variation explained by the host principal components was 3.8%, higher than expected by chance alone (empirical *p* = 0.0001; Fig. 3b). The distribution of empirical *p* values for the *R*^2^ values of the individual species was strongly shifted towards zero (Fig. 3c), indicating that strong correlations were not limited to a few species, but that genetic population structure influenced overall microbial configurations. We observed a similar effect on the species level in oral sites. In buccal mucosa, the genomic principal components described on average 5.2% (empirical *p* = 0.0008) of the species-level variation; in tongue dorsum, this figure was 4.1% (empirical *p* = 0.0034). In an identical analysis of MetaCyc metabolic pathway abundance, we found only the pathways in the gut microbiomes to be significantly correlated with common variant principal components. In summary, the association between high-level host genetic features and microbiome properties was significant at multiple body sites.

When examining the correlation of individual microbial features with host genetics that contributed to these averages, certain features showed much stronger individual associations. In stool, where the genetic correlation was the strongest, five species out of 118 were significantly associated (false discovery rate (FDR) < 0.05 by permutation test), with *R*^2^ values reaching almost 10% (Fig. 3c). Of these five species, *Lachnospiraceae bacterium*, *Roseburia intestinalis*, and *Subdoligranulum* (unclassified) were all positively correlated with the first genomic principal component, demonstrating that these species have higher abundance in donors of Caucasian origin. Another significant species, *Sutterella wadsworthensis*, was associated with PC4, which separates donors of Asian origin into two groups. Examining other body sites, we found that *Porphyromonas catoniae*, *Propionibacterium propionicum*, and unclassified *Gemella* were significantly associated with host genomic variation in buccal mucosa (Additional file 1: Figure S4 and Table S1).

A similar pathway-level analysis revealed a large number (82 of 541) of pathways significantly (FDR < 0.05 as above) correlated with genetic principal components in stool (Fig. 3c). Several pathways were related to amino acid and short-chain fatty acid biosynthesis and degradation. In a more systematic view, we found that the members of the fermentation superclass of the MetaCyc database were significantly enriched in the top pathway rankings (Fig. 3c). Most of these pathways were associated with the first genetic principal component that distinguishes white donors from other racial or ethnic ancestries. Such functional enrichments can point to ethnic differences in diet, but also to genetic variability in the ability to metabolize certain nutrients.

In other body sites, pathway-level variability was on average not correlated with genetic principal components, although some individual correlated pathways were found (Additional file 1: Figure S5 and Table S1). For example, a number of pathways in tongue dorsum microbiomes correlated strongly with genetic principal components. Interestingly, almost all of the associated pathways were related to respiration and the tricarboxylic acid (TCA) cycle, indicative of an oxygen gradient and differences in aerobic respiration by oral organisms of the tongue dorsum between donors. The enrichment of the TCA cycle in the oral microbiome and fermentation in the gut microbiome reflect the dominant metabolic features of the corresponding microbiomes and show how these can be affected by host genetics and environmental factors correlated with genetic ancestry.

### Related donors have similar microbiomes

Although the HMP cohort included donors related to each other, this information was not available in the collected metadata. Genomic sequencing of the donors allowed us to infer the extent of relation between all donor pairs and identify up to third degree relatives among them. Using common SNVs (MAF > 0.05) for the analysis, we identified 11 pairs of first degree relatives and one pair of third degree relatives.

**Fig. 4:**
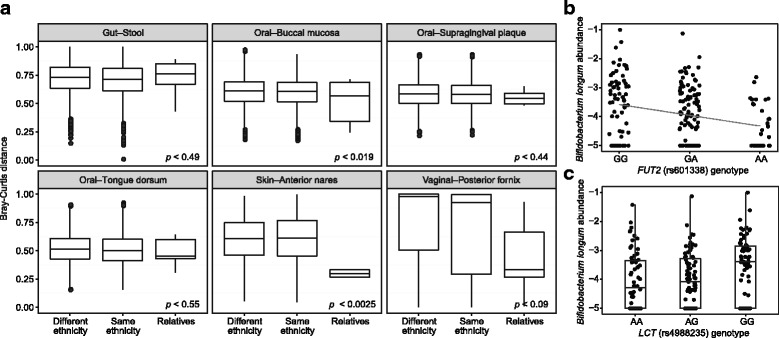
Kinship and microbiome similarity and replication of known associations. **a** Bray-Curtis similarity between the 12 pairs of close relatives (third degree or closer) identified from genetic data compared to similarities between other pairs. The *p* values correspond to results of *t* tests between similarity scores for relatives, against all other pairs. **b** Association between *FUT2* secretor variant and *B. longum.* **c** Association between genetic variant rs4988235 near the *LCT* gene and *B. longum*. In both **b** and **c** we display log_10_ transformed relative abundance

We next sought to determine whether the degree of relation was reflected in the similarity of their microbiomes. For this analysis we calculated the Bray-Curtis distance between all donor pairs and divided the pairs into three groups: same ethnicity, different ethnicity, and relatives (Fig. 4a). As could be expected from the PCA, the samples within ethnic groups were on average slightly more similar than samples from different ethnic groups, but microbiome similarity between related donors was more pronounced. With the exception of the gut, in all tested body sites, microbiome community composition between relatives were more similar than between random donor pairs; in anterior nares and buccal mucosa, the effect was also statistically significant by *t* test between unrelated and related similarity scores. For vaginal samples the effect was also pronounced, but we did not have enough female-female pairs to achieve statistical significance.

### *FUT2* and *LCT* genotypes are associated with *Bifidobacterium longum*

To study the influence of individual genetic variants on microbiome composition, we began with known associations of *FUT2* and *LCT. FUT2* is responsible for the transfer of terminal fucose residues to mucosal glycans [48]. Bifidobacteria also use mucus-derived fucose as a carbon source, and abundance and diversity of *B. longum* is significantly lower in non-secretors (individuals with a premature stop codon in *FUT2*, rs601338) compared to secretors [27].

To determine whether this association could be verified in the HMP cohort, we searched for microbial species correlated with the host fucosyltransferase secretor genotype (MAF = 0.41). We used linear regression to predict the relative abundance of each individual species based on the secretor genotype dosage. *B. longum* had the strongest correlation of the 118 tested species (FDR = 0.018; Fig. 4b), with increased relative abundance in the secretor genotype relative to the non-secretor genotype. This finding is consistent with previous experimental observations and demonstrates that the cohort is sufficiently powered to validate targeted microbial-host association hypotheses.

Similarly, *LCT* has been associated with increased abundance of lactose-metabolizing Bifidobacteria in the gut [22, 25]. *LCT* encodes lactase, the enzyme responsible for breaking down lactose in the upper GI tract; in tandem with increased Bifidobacteria, this suggests that more dietary lactose collects in the large intestine. The ability to produce lactase in adulthood or lactose intolerance (hypolactasia) is controlled by the presence of a homozygous G allele in rs4988235 SNV close to *LCT* [49]. A recent finding that milk consumption and Bifidobacteria abundance is positively correlated only in people with the hypolactasia gene variant [26] supports this mechanism.

In the HMP cohort, we compared bacterial species abundances in stool between donors with the hypolactasia and alternative variants. Because hypolactasia is a recessive trait, we used a *t* test to compare the 64 donors with the homozygous G genotype to the rest of the 145 donors. After testing each of the 118 individual species abundance against the presence of this variant, we found that *B. longum* had the strongest effect (FDR = 0.095), thereby confirming the previously found association (Fig. 4c).

### Microbial associations with host genome variants are body site-specific

Finally, we assessed associations between host genome and microbiome variation in a non-targeted manner directly through a genome-wide association study. We performed the analysis separately for each body site, concentrating on SNVs with MAF > 0.1 and comparing them to both microbial species- and metabolic pathway-level abundances. We used ordinary linear regression models, taking into account the effects of sex, ethnicity, and sample collection location. After filtering the microbial features (see Methods), we identified 120–160 species in GI tract and oral samples and approximately 25 species in skin and vaginal samples. The number of metabolic pathways passing filtering was considerably higher, between 350 and 530 major pathways per site. Together the large numbers of SNVs, body sites, and microbial features in the analysis impose a strict significance criterion (*p* < 3 × 10^–12^ according to Bonferroni correction for multiple testing), which, in combination with our modest sample size, limits our discovery potential to associations with very large effect sizes.

For this reason, we first limited our analysis to SNVs found in the National Human Genome Research Institute (NHGRI) Genome-Wide Association Studies (GWAS) Catalog [50], hypothesizing that these SNVs were enriched with genomic variants that have potential impact on microbiome properties. This set included SNVs associated with a diverse set of quantitative traits ranging from complex diseases to anthropometric measurements. A total of 16,869 of these SNVs were found in our data, but we did not detect any significant associations using this subset of SNVs. Furthermore, according to the quantile-quantile plot of the comparisons, there was no systematic enrichment of smaller *p* values among the comparisons (Additional file 1: Figures S6 and S7). We did not obtain significant results with even more constrained variant sets associated with inflammatory bowel disease or with any of the high-level GWAS Catalog subcategories (e.g., “immune system disorders,” “digestive system”).

We next ran the association analysis on all common SNVs. We did not see any associations with *p* values smaller than multiple testing-corrected significance limits. However, there were a number of associations (Additional file 1: Figures S8 and S9 and Table S2) with relatively small *p* values. These associations form a rich source of information for future microbiome-wide association studies and investigators interested in specific genes or microbial species.

Because of the unique design of the HMP cohort, we were also able to investigate the nature of the associations between body sites. Studies in expression quantitative trait loci have reported remarkable stability of gene expression-genotype associations across tissues [51]. Here, we did not observe any SNV-microbe pairs in the top of the association rankings for multiple body sites, demonstrating the more indirect nature of these associations as well as the unique community construction principles for each body site. When considering the distribution of the best *p* values from each body site irrespective of the associated organism, we still could not find any SNVs that were more strongly associated with microbes than expected by chance. This finding further suggests that the genetic mechanisms behind microbiome composition are body site-specific.

## Discussion

Previous studies of the human microbiome have revealed that microbiomes can be remarkably different between individuals, while their composition within an individual remains relatively stable over time. These observations suggest that genetic factors may be one influence on microbiome composition, in addition to recognized ecological and environmental factors such as colonization, diet, medications, and lifestyle. Although studies in twins and mice have suggested some genetic influence on microbiome composition, reports of direct associations are sparse. The main obstacle for such discovery studies is the lack of large well-described cohorts with both microbial abundance and genotype measurements. Here, we report host whole genome sequencing for the HMP cohort, one of the largest and most comprehensively microbially characterized populations in the world.

Host whole-genome sequencing provides the opportunity to associate host genetic variation with microbial features in this cohort. We found that most microbes are correlated to genetic principal components, especially in stool, but also in oral samples. Similar approaches have been applied on subsets of the HMP cohort using haplogroups [30] and common variants [25]. Both of these studies identified associations between high-level genetic features and various microbiome features; however, the mechanistic bases of those associations remain unclear. Some associations are likely to arise from cultural differences in diet or behavior, but human populations also vary in their ability to digest certain nutrients and can thereby create specific microenvironments for gut microbes.

The combination of host and microbiome sequencing in the HMP cohort is unique, inasmuch as it represents the first non-disease population with body-wide metagenomics and deep human whole-genome sequencing. While the ~ 300 individuals of the HMP (~100 with shotgun metagenomics) are of course not enough for a typical association discovery study, the example applications we investigate here are representative of how this data resource might be used in the future. In combination with larger, targeted populations, for example, the HMP cohort can now be used as a baseline, comparator, or validation in microbiome-genetic association studies at most body sites of interest. The high-quality host and microbial data here are appropriate for future meta-analyses and as a methodological framework to make larger discovery efforts more efficient and less costly.

Previous comparisons between monozygotic and dizygotic twins [22, 52] have identified a set of microbial taxa with higher than expected heritability coefficients. We examined the behavior of these organisms in our cohort. Unfortunately, not all of the taxa with the highest heritability coefficients were present in our data, likely due to differences in the biases introduced by 16S and metagenomic sequencing and subsequent processing. We did apply principal component correlation analysis to the nine heritable taxa that were present in the HMP cohort; however, none were significantly correlated with genetic principal components.

Microbial communities are highly adapted to the environment of particular body locations [1]. We found that associations between microbial and genetic features, on both the host principal component and single-variant levels, were not shared by the microbiomes between body sites. Therefore, association studies within different body sites have the potential to uncover distinct genetic mechanisms influencing biogeographically distinct microbial features. The HMP cohort, with its broad sampling of microorganisms across five major body regions, represents a unique resource for such studies. While the number of studies concentrating on the GI tract microbiome is increasing [20, 22–24, 26], the microbial ecologies of other body sites have been studied to a significantly lesser degree.

In our genome-wide association analysis, no individual associations reached the threshold for statistical significance. This is contrast with the findings of Blekhman et al. [25], who reported 83 significant associations using “contaminant” human reads from a subset of HMP participants’ metagenomes. The main reason for the discrepancy is the choice of significance thresholds. Blekhman et al. used FDR multiple hypothesis test correction with a threshold of 0.1, whereas we used a more stringent Bonferroni correction. We felt this to be more appropriate to avoid inflation of FDR values in what is, overall, a fairly small population, particularly due to the numerical properties of the genetic (e.g., linkage disequilibrium) and microbial (e.g., zero inflation and non-normal distributions) data. Without this assumption and using the FDR < 0.1 control instead, we see a number of associations across body sites (Additional file 1: Table S2). However, the statistical significance of these associations was not confirmed in subsequent permutation testing.

Nevertheless, many findings between the two datasets were qualitatively similar, including an association between *LCT* variants and *Bifidobacterium* abundance that has also been validated in additional external studies [22, 25, 26]. Highly ranked associations from our tests may be similarly relevant findings, such as variants near *SULT2B1* that are relatively strongly associated with *Actinomyces viscocus* in skin (*p* = 2.4 × 10^–8^). This gene is involved in processing dehydroepiandrosterone, a hormone implicated in epidermal thickness and sebum production of the skin [53]. In addition, the endoplasmic reticulum aminopeptidase 1 (*ERAP1)* gene, which is involved in antigen presentation and associated with inflammatory bowel disease [54] and a number of other autoimmune diseases [55–57], appeared in two associations. In buccal mucosa samples, *ERAP1* was associated with *Actinomyces graevenitzii* (*p* = 3.9 × 10^–8^), a normal member of the oral microflora that can, under some circumstances, act as an opportunistic pathogen and cause pulmonary abscesses [58]. In the GI tract, different variants close to *ERAP1* were associated with *Lachnospiraceae* bacteria (*p* = 3.2 × 10^–8^). Interestingly, *Lachnospiraceae* was the most differentially regulated microbial family in the terminal ileum of patients with ankylosing spondylitis [59], a disease associated with polymorphisms in *ERAP1* [60]. These and other putative associations present compelling evidence, but will have to be confirmed by future studies.

In general, to reliably identify genetic influences on microbial features, it will be necessary to increase the number of samples with both host and microbial sequencing. This is particularly true when combining baseline “healthy” and disease-specific populations, genetic variants, and microbial variants. For example, based on the *NOD2*-*Enterobacteriaceae* association found in patients with inflammatory bowel disease, it was calculated that detecting the same association in a genome-wide significant manner would require 3700 patients [24]. In this regard, two recent papers [23, 26] emphasize how good validation cohorts can help to filter results from otherwise underpowered studies.

Combining HMP data with data from other cohorts through meta-analysis or as a validation cohort is thus one way to facilitate future studies. By validating two previous genotype-microbe associations (*FUT2* and *LCT*), we showed that the HMP genetic data provide power to validate targeted hypotheses and contribute evidence to meta-analyses. Given that the HMP features broad biogeographic microbial sampling across the body, and the human sequencing data are of high quality and consistent with data from projects such as GoNL and 1000 Genomes, these data are well suited for such purposes. The comprehensive nature of whole-genome sequencing makes the data versatile, allowing the combination of HMP data with genome analysis technologies from genotyping arrays for common variants to exome sequencing for coding variation or other targeted approaches.

## Conclusions

Here, we present the results of whole-genome sequencing of donors from the HMP healthy cohort study, enabling the study of host genetic effects on the microbiome properties of multiple body sites in this cohort. We detected significant correlations between high-level genetic features and both microbial species and community functional profiles. Using these data, we verified that variants near the *LCT* and *FUT2* genes associated significantly with the abundance of *B. longum* in stool. In a broader, untargeted genome-wide setting, we did not identify significant associations with single variants, mainly due to stringent multiple testing criteria imposed by the numbers of microbial features and body sites in combination with a relatively modest sample size. The top associations, however, provide an initial picture of body-wide host-microbial interaction potential. In addition, the dataset as a whole, when paired with the comprehensive microbiome sequencing already performed on this cohort, constitutes an invaluable resource for further studies.

## Additional file

Additional file 1: Figure S1. Sequencing and variant call quality metrics. A. Percentage of contamination and chimeric reads. B. Various metrics based on variant calling. **Figure S2.** Distribution of genic and intergenic variants. **Figure S3.** Combined PCA between 1000 Genomes and HMP300. **Figure S4.** Correlation between high-level genetic features and microbial species in non-gut body sites. **Figure S5.** Correlation between high-level genetic features and microbial metabolic pathways in non-gut body sites. **Figure S6.** Quantile-quantile plots for association analysis between microbial species and GWAS Catalog SNVs. **Figure S7.** Quantile-quantile plots for association analysis between microbial metabolic pathways and GWAS Catalog SNVs. **Figure S8.** Putative SNV-microbial species associations. **Figure S9.** Putative SNV-microbial metabolic pathway associations. **Table S1.** Raw statistics for genetic principal component analysis. **Table S2.** Top SNV and microbiome association results. (PDF 1953 kb)

## Abbreviations

AC: Allele count
FDR: False discovery rate
GI: Gastrointestinal
GoNL: Genome of the Netherlands
GWAS: Genome-Wide Association Study
HMP: Human Microbiome Project
LoF: Loss of function
MAF: Minor allele frequency
NHGRI: National Human Genome Research Institute
PCA: Principal component analysis
SNV: Single nucleotide variant
VEP: Variant Effect Predictor
WMS: Whole metagenome sequencing
