# Supplementary material for: Host genetic variation and its microbiome interactions within the Human Microbiome Project
Source: Genome Med. 2018 Jan 29;10:6. doi: 10.1186/s13073-018-0515-8 (PMC5789541; doi:10.1186/s13073-018-0515-8)
Supplement: Additional file 1: Figure S1. — Sequencing and variant call quality metrics. A. Percentage of contamination and chimeric reads. B. Various metrics based on variant calling. Figure S2. Distribution of genic and intergenic variants. Figure S3. Combined PCA between 1000 Genomes and HMP300. Figure S4. Correlation between high-level genetic features and microbial species in non-gut body sites. Figure S5. Correlation between high-level genetic features and microbial metabolic pathways in non-gut body sites. Figure S6. Quantile-quantile plots for association analysis between microbial species and GWAS Catalog SNVs. Figure S7. Quantile-quantile plots for association analysis between microbial metabolic pathways and GWAS Catalog SNVs. Figure S8. Putative SNV-microbial species associations. Figure S9. Putative SNV-microbial metabolic pathway associations. Table S1. Raw statistics for genetic principal component analysis. Table S2. Top SNV and microbiome association results. (PDF 1953 kb) [file 13073_2018_515_MOESM1_ESM.pdf]

Supplemental Figure and Table legends

Figure S1. Sequencing and variant call quality metrics. **a** Percentage of contamination and chimeric reads. **b** Various metrics based on variant calling.

Figure S2. Distribution of genic and intergenic variants. Proportion of intronic, exonic and intergenic variants in HMP300, GoNL, and 1000 Genome cohorts.

Figure S3. Combined PCA analysis between 1000 Genomes and HMP300. Both panels correspond to the same underlying coordinate system defined by a PCA of a merger of common variants from 1000 Genomes and HMP300 datasets.

Figure S4. Correlation between high-level genetic features and microbial species in non-gut body sites. Same analysis as in Figure 2b, performed using other body sites.

Figure S5. Correlation between high-level genetic features and microbial metabolic pathways in non-gut body sites. Same analysis as in Figure 2c, performed using other body sites.

Figure S6. Quantile-quantile plots for association analysis between microbial species and GWAS catalog SNVs.

Figure S7. Quantile-quantile plots for association analysis between microbial metabolic pathways and GWAS catalog SNVs.

Figure S8. Putative SNV–microbial species associations. All associations between single nucleotide variants and microbial species with $p < 5 \times 10^{-8}$ are shown, with color and size of the edges displaying sampling site and association strength respectively.

Figure S9. Putative SNV–microbial metabolic pathway associations. All associations between single nucleotide variants and microbial metabolic pathways with $p < 5 \times 10^{-8}$ are shown, with color and size of the edges displaying sampling site and association strength respectively.

Table S1. Raw statistics for genetic principal component analysis. Sheet 1 contains statistics for average R^2 calculations shown in Figure 2b. The additional 12 sheets provide statistics for individual species- and pathway-level analyses for all six body sites, corresponding to Figure 2c and Figures S4-S5.

Table S2. Top SNV and microbiome association results. Top associations ($p < 10^{-6}$) are shown for both species and pathways in all six body sites.

Figure S1

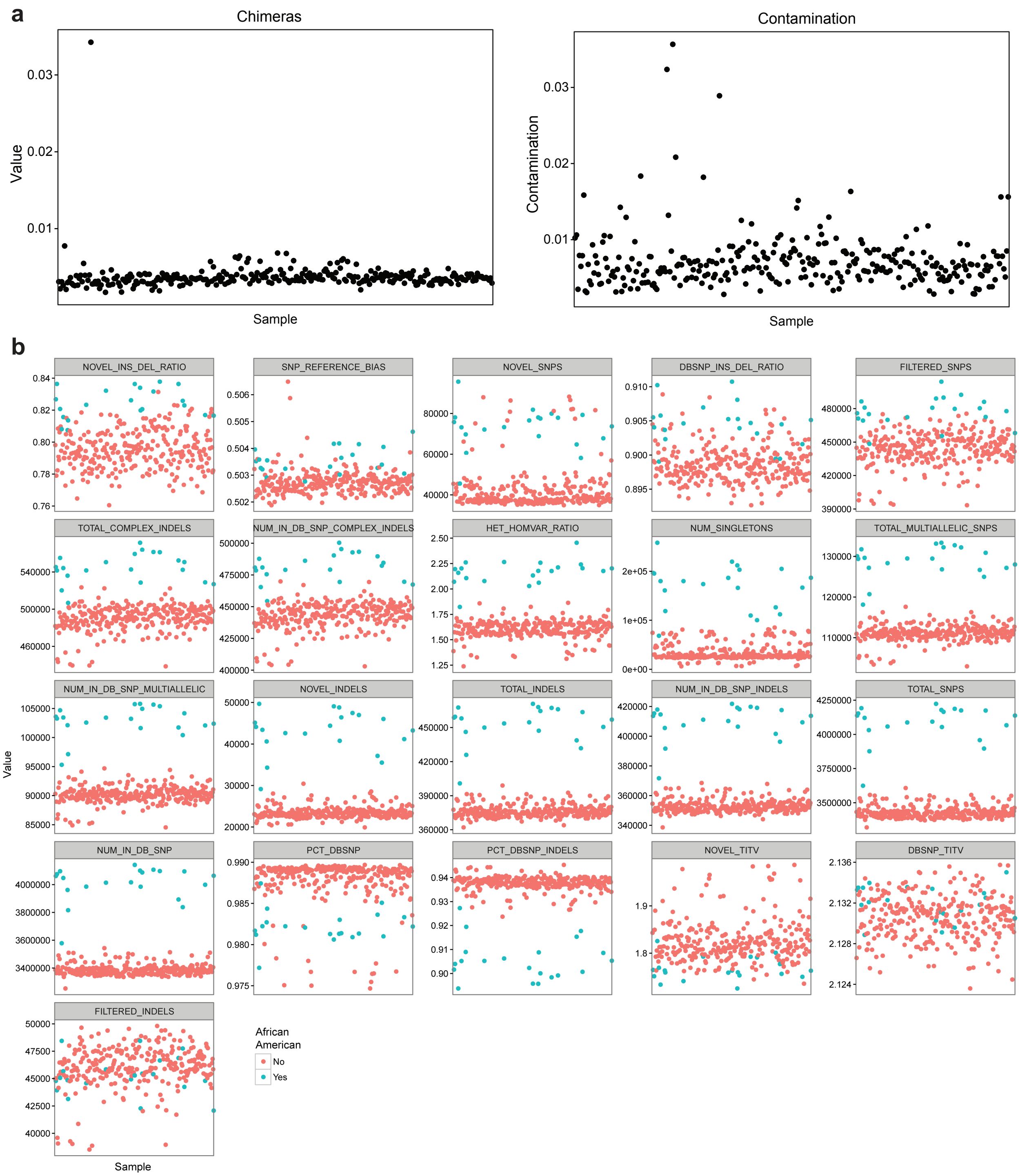
a
Chimeras
Value
Sample
Contamination
Contamination
Sample
b
NOVEL_INS_DEL_RATIO
SNP_REFERENCE_BIAS
NOVEL_SNPS
DBSNP_INS_DEL_RATIO
FILTERED_SNPS
TOTAL_COMPLEX_INDELS
NUM_IN_DB_SNP_COMPLEX_INDELS
HET_HOMVAR_RATIO
NUM_SINGLETONS
TOTAL_MULTIALLELIC_SNPS
NUM_IN_DB_SNP_MULTIALLELIC
NOVEL_INDELS
TOTAL_INDELS
NUM_IN_DB_SNP_INDELS
TOTAL_SNPS
NUM_IN_DB_SNP
PCT_DBSNP
PCT_DBSNP_INDELS
NOVEL_TITV
DBSNP_TITV
FILTERED_INDELS
Value
Sample
African American
No
Yes

Figure S2

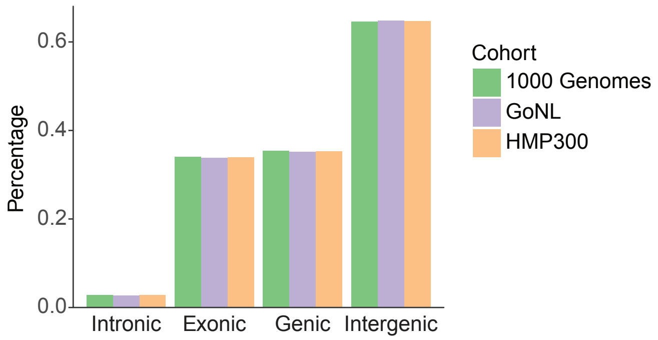

Percentage
0.6
0.4
0.2
0.0
Intronic
Exonic
Genic
Intergenic
Cohort
1000 Genomes
GoNL
HMP300

Figure S3

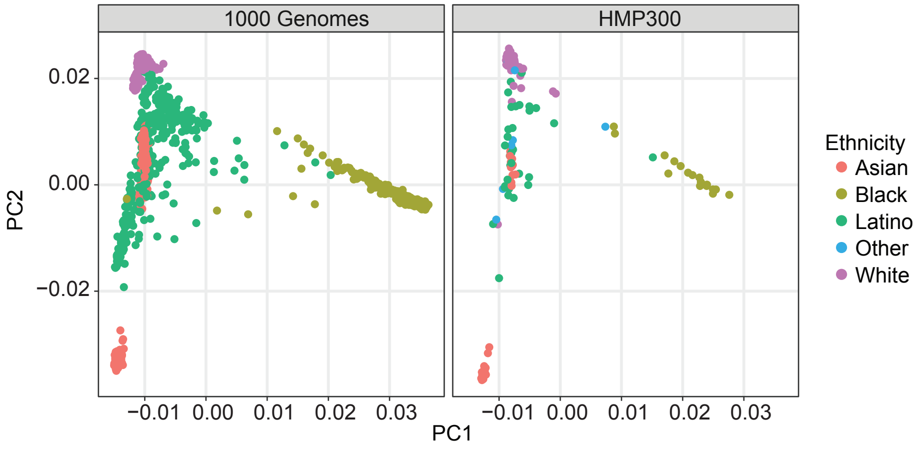
1000 Genomes
HMP300
0.02
0.00
−0.02
PC2
−0.01 0.00 0.01 0.02 0.03
−0.01 0.00 0.01 0.02 0.03
PC1
Ethnicity
Asian
Black
Latino
Other
White

Figure S4

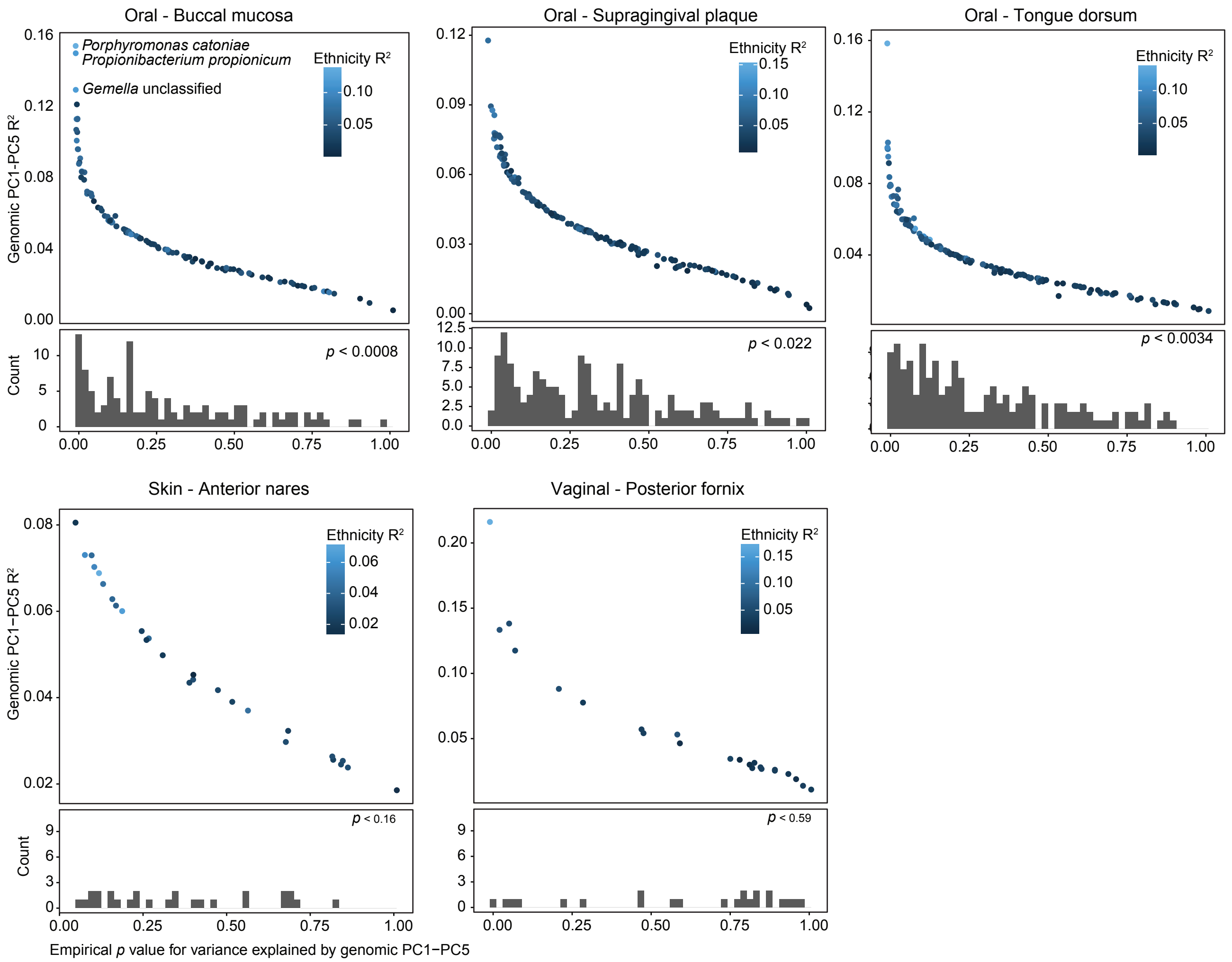

Oral - Buccal mucosa
Porphyromonas catoniae
Propionibacterium propionicum
Gemella unclassified
Ethnicity R²
Genomic PC1-PC5 R²
Count
p < 0.0008
Oral - Supragingival plaque
p < 0.022
Oral - Tongue dorsum
p < 0.0034
Skin - Anterior nares
p < 0.16
Vaginal - Posterior fornix
p < 0.59
Empirical p value for variance explained by genomic PC1−PC5

Figure S5

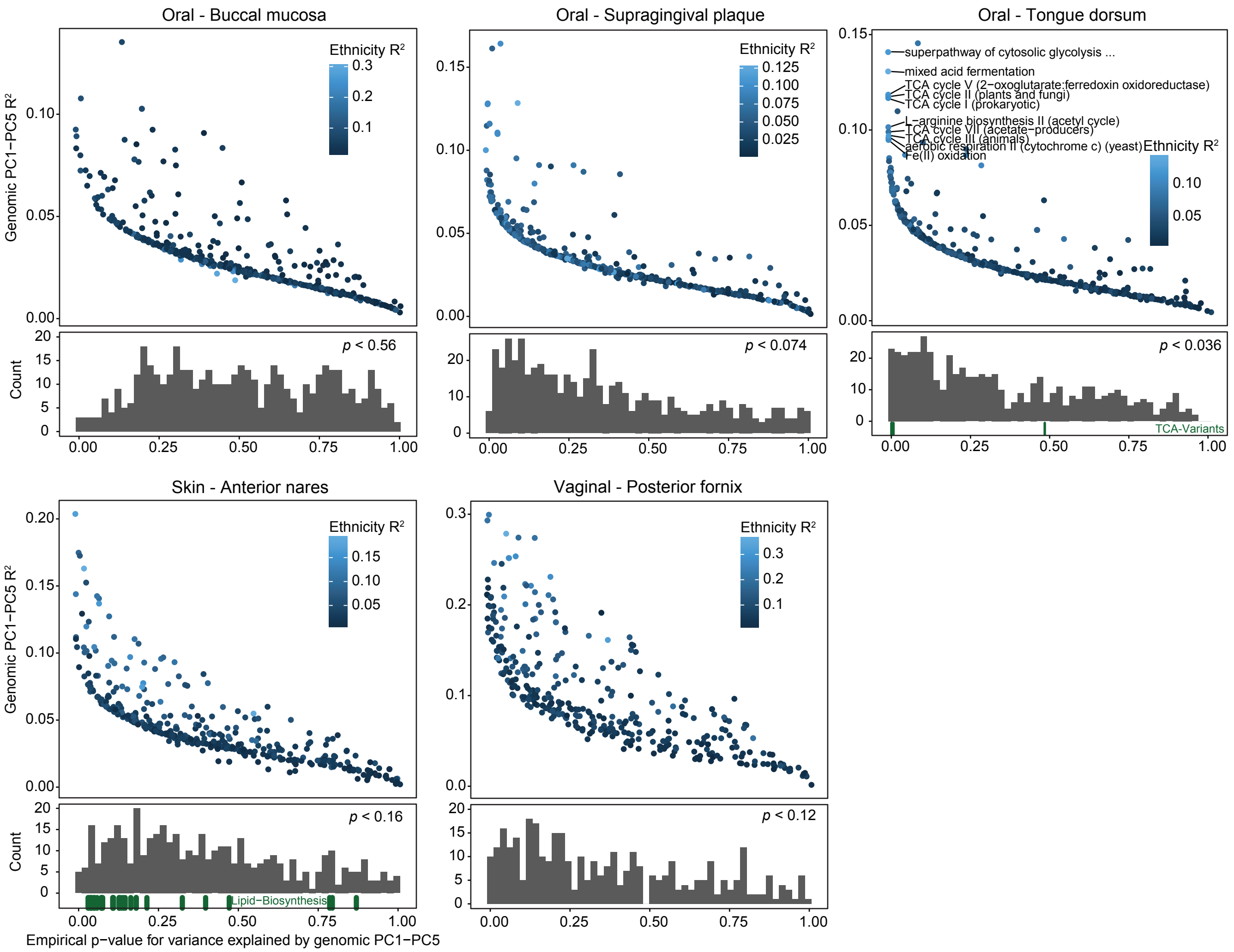
Oral - Buccal mucosa
Oral - Supragingival plaque
Oral - Tongue dorsum
Skin - Anterior nares
Vaginal - Posterior fornix
Ethnicity R²
Genomic PC1−PC5 R²
Count
p < 0.56
p < 0.074
p < 0.036
p < 0.16
p < 0.12
superpathway of cytosolic glycolysis ...
mixed acid fermentation
TCA cycle V (2−oxoglutarate:ferredoxin oxidoreductase)
TCA cycle II (plants and fungi)
TCA cycle I (prokaryotic)
L−arginine biosynthesis II (acetyl cycle)
TCA cycle VII (acetate−producers)
TCA cycle III (animals)
aerobic respiration II (cytochrome c) (yeast)
Fe(II) oxidation
TCA-Variants
Lipid−Biosynthesis
Empirical p−value for variance explained by genomic PC1−PC5

Figure S6

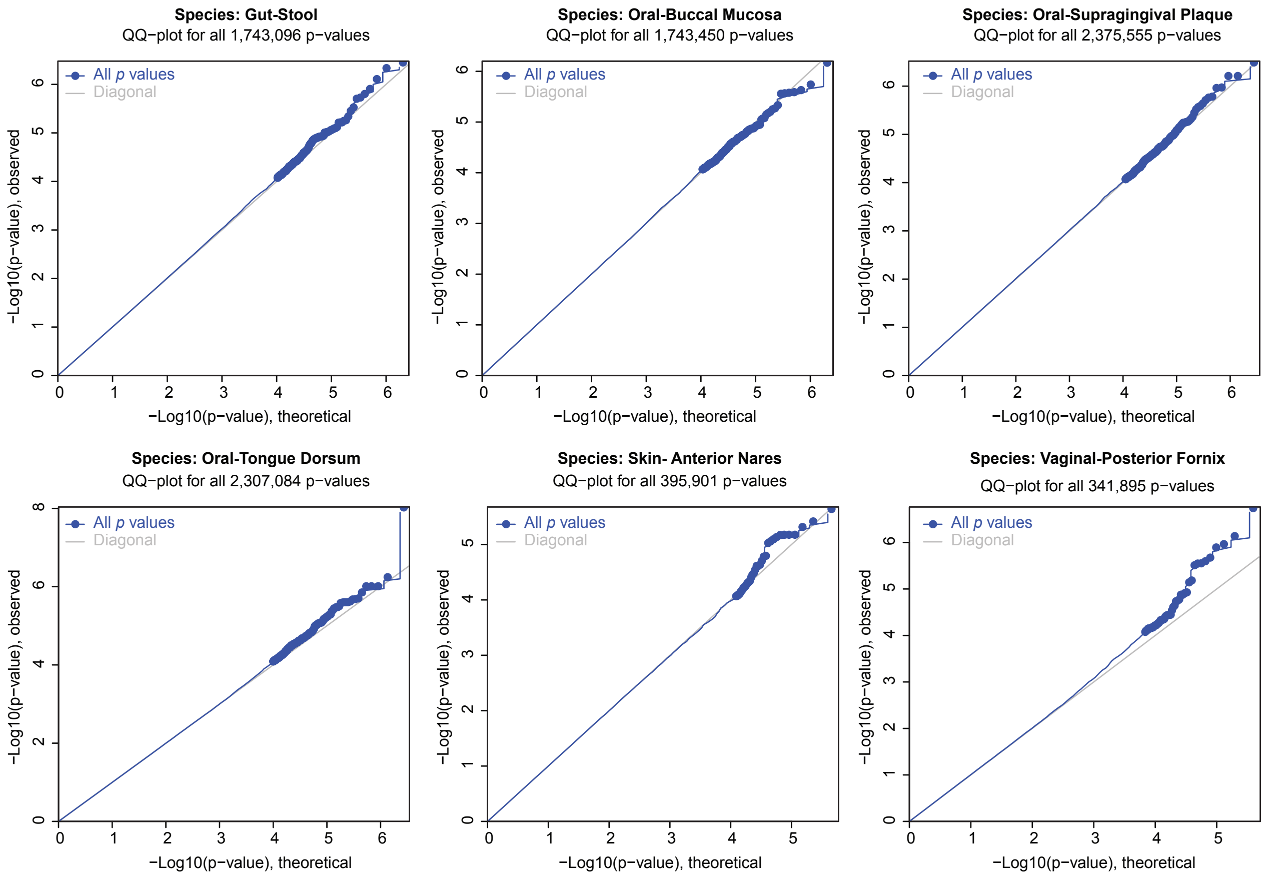
Species: Gut-Stool
QQ−plot for all 1,743,096 p−values
Species: Oral-Buccal Mucosa
QQ−plot for all 1,743,450 p−values
Species: Oral-Supragingival Plaque
QQ−plot for all 2,375,555 p−values
Species: Oral-Tongue Dorsum
QQ−plot for all 2,307,084 p−values
Species: Skin- Anterior Nares
QQ−plot for all 395,901 p−values
Species: Vaginal-Posterior Fornix
QQ−plot for all 341,895 p−values
All p values
Diagonal
−Log10(p−value), observed
−Log10(p−value), theoretical

Figure S7

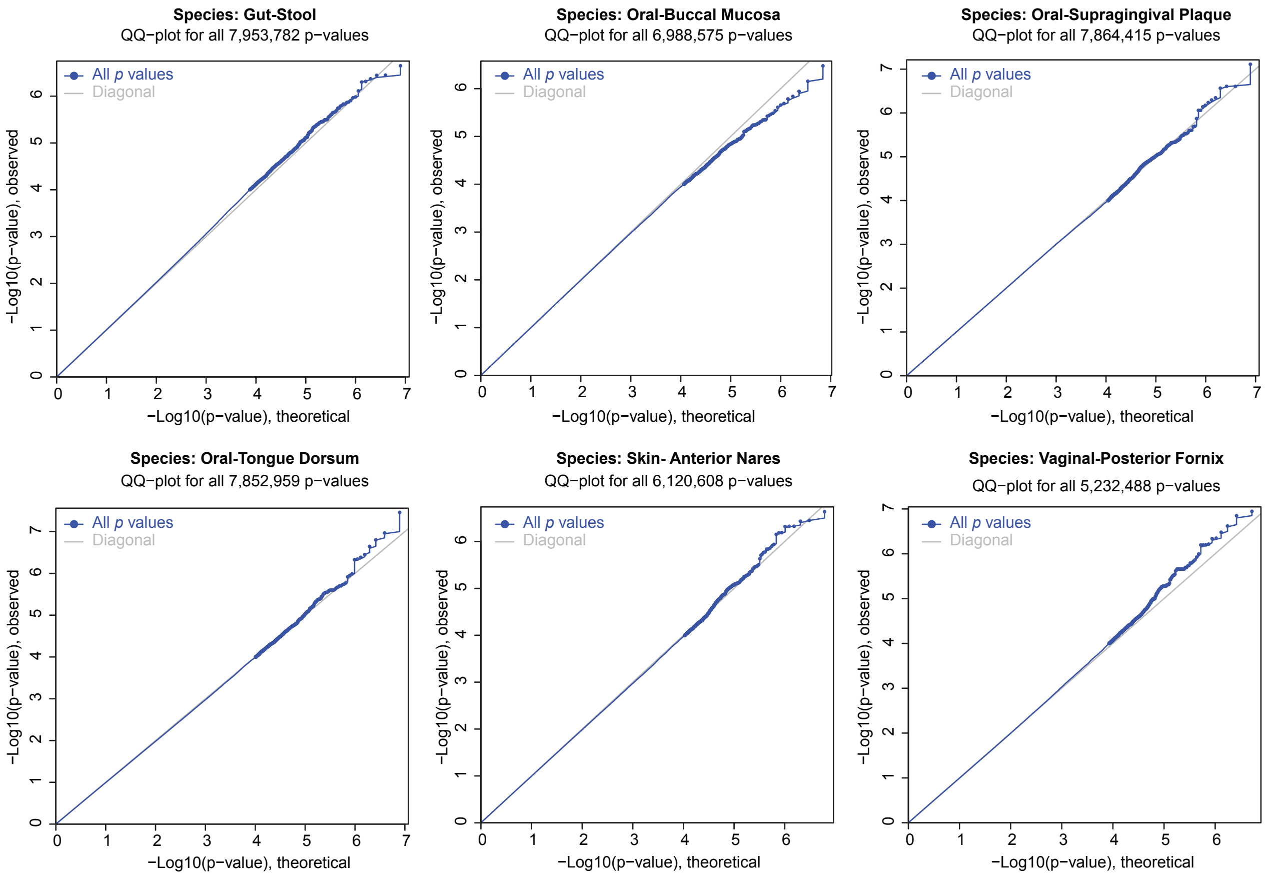
Species: Gut-Stool
QQ−plot for all 7,953,782 p−values
Species: Oral-Buccal Mucosa
QQ−plot for all 6,988,575 p−values
Species: Oral-Supragingival Plaque
QQ−plot for all 7,864,415 p−values
Species: Oral-Tongue Dorsum
QQ−plot for all 7,852,959 p−values
Species: Skin- Anterior Nares
QQ−plot for all 6,120,608 p−values
Species: Vaginal-Posterior Fornix
QQ−plot for all 5,232,488 p−values
All p values
Diagonal
−Log10(p−value), observed
−Log10(p−value), theoretical

Figure S8

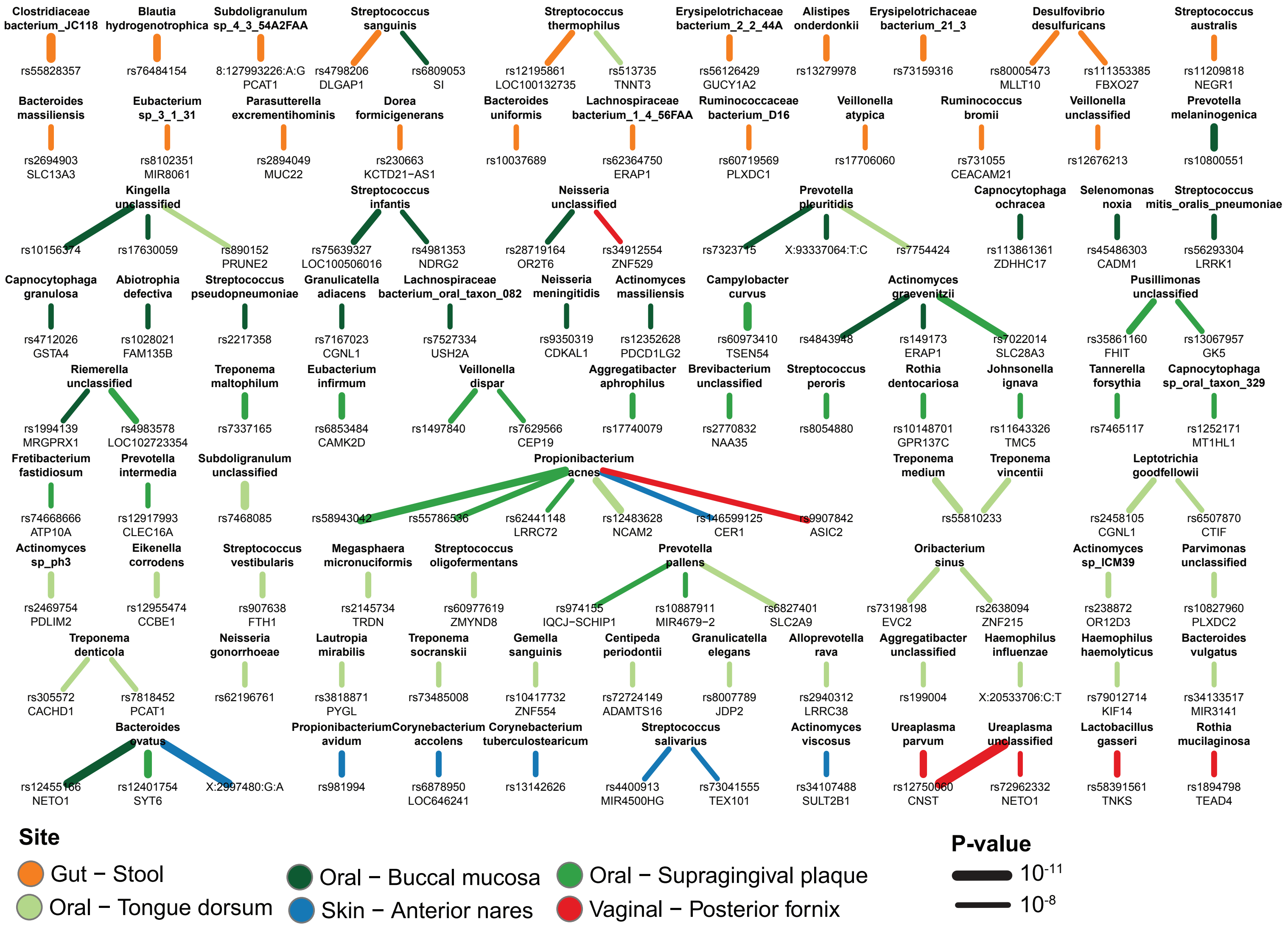

Clostridiaceae bacterium_JC118
rs55828357
Blautia hydrogenotrophica
rs76484154
Subdoligranulum sp_4_3_54A2FAA
8:127993226:A:G PCAT1
Streptococcus sanguinis
rs4798206 DLGAP1
rs6809053 SI
Streptococcus thermophilus
rs12195861 LOC100132735
rs513735 TNNT3
Erysipelotrichaceae bacterium_2_2_44A
rs56126429 GUCY1A2
Alistipes onderdonkii
rs13279978
Erysipelotrichaceae bacterium_21_3
rs73159316
Desulfovibrio desulfuricans
rs80005473 MLLT10
rs111353385 FBXO27
Streptococcus australis
rs11209818 NEGR1
Bacteroides massiliensis
rs2694903 SLC13A3
Eubacterium sp_3_1_31
rs8102351 MIR8061
Parasutterella excrementihominis
rs2894049 MUC22
Dorea formicigenerans
rs230663 KCTD21−AS1
Bacteroides uniformis
rs10037689
Lachnospiraceae bacterium_1_4_56FAA
rs62364750 ERAP1
Ruminococcaceae bacterium_D16
rs60719569 PLXDC1
Veillonella atypica
rs17706060
Ruminococcus bromii
rs731055 CEACAM21
Veillonella unclassified
rs12676213
Prevotella melaninogenica
rs10800551
Kingella unclassified
rs10156374
rs17630059
rs890152 PRUNE2
Streptococcus infantis
rs75639327 LOC100506016
rs4981353 NDRG2
Neisseria unclassified
rs28719164 OR2T6
rs34912554 ZNF529
Prevotella pleuritidis
rs7323715
X:93337064:T:C
rs7754424
Capnocytophaga ochracea
rs113861361 ZDHHC17
Selenomonas noxia
rs45486303 CADM1
Streptococcus mitis_oralis_pneumoniae
rs56293304 LRRK1
Capnocytophaga granulosa
rs4712026 GSTA4
Abiotrophia defectiva
rs1028021 FAM135B
Streptococcus pseudopneumoniae
rs2217358
Granulicatella adiacens
rs7167023 CGNL1
Lachnospiraceae bacterium_oral_taxon_082
rs7527334 USH2A
Neisseria meningitidis
rs9350319 CDKAL1
Actinomyces massiliensis
rs12352628 PDCD1LG2
Campylobacter curvus
rs60973410 TSEN54
Actinomyces graevenitzii
rs4843948
rs149173 ERAP1
rs7022014 SLC28A3
Pusillimonas unclassified
rs35861160 FHIT
rs13067957 GK5
Riemerella unclassified
rs1994139 MRGPRX1
rs4983578 LOC102723354
Treponema maltophilum
rs7337165
Eubacterium infirmum
rs6853484 CAMK2D
Veillonella dispar
rs1497840
rs7629566 CEP19
Aggregatibacter aphrophilus
rs17740079
Brevibacterium unclassified
rs2770832 NAA35
Streptococcus peroris
rs8054880
Rothia dentocariosa
rs10148701 GPR137C
Johnsonella ignava
rs11643326 TMC5
Tannerella forsythia
rs7465117
Capnocytophaga sp_oral_taxon_329
rs1252171 MT1HL1
Fretibacterium fastidiosum
rs74668666 ATP10A
Prevotella intermedia
rs12917993 CLEC16A
Subdoligranulum unclassified
rs7468085
Propionibacterium acnes
rs58943042
rs55786536
rs62441148 LRRC72
rs12483628 NCAM2
rs146599125 CER1
rs9907842 ASIC2
Treponema medium
Treponema vincentii
rs55810233
Leptotrichia goodfellowii
rs2458105 CGNL1
rs6507870 CTIF
Actinomyces sp_ph3
rs2469754 PDLIM2
Eikenella corrodens
rs12955474 CCBE1
Streptococcus vestibularis
rs907638 FTH1
Megasphaera micronuciformis
rs2145734 TRDN
Streptococcus oligofermentans
rs60977619 ZMYND8
Prevotella pallens
rs974155 IQCJ−SCHIP1
rs10887911 MIR4679−2
rs6827401 SLC2A9
Oribacterium sinus
rs73198198 EVC2
rs2638094 ZNF215
Actinomyces sp_ICM39
rs238872 OR12D3
Parvimonas unclassified
rs10827960 PLXDC2
Treponema denticola
rs305572 CACHD1
rs7818452 PCAT1
Neisseria gonorrhoeae
rs62196761
Lautropia mirabilis
rs3818871 PYGL
Treponema socranskii
rs73485008
Gemella sanguinis
rs10417732 ZNF554
Centipeda periodontii
rs72724149 ADAMTS16
Granulicatella elegans
rs8007789 JDP2
Alloprevotella rava
rs2940312 LRRC38
Aggregatibacter unclassified
rs199004
Haemophilus influenzae
X:20533706:C:T
Haemophilus haemolyticus
rs79012714 KIF14
Bacteroides vulgatus
rs34133517 MIR3141
Bacteroides ovatus
rs12455166 NETO1
rs12401754 SYT6
X:2997480:G:A
Propionibacterium avidum
rs981994
Corynebacterium accolens
rs6878950 LOC646241
Corynebacterium tuberculostearicum
rs13142626
Streptococcus salivarius
rs4400913 MIR4500HG
rs73041555 TEX101
Actinomyces viscosus
rs34107488 SULT2B1
Ureaplasma parvum
rs12750060 CNST
Ureaplasma unclassified
rs72962332 NETO1
Lactobacillus gasseri
rs58391561 TNKS
Rothia mucilaginosa
rs1894798 TEAD4
Site
Gut − Stool
Oral − Tongue dorsum
Oral − Buccal mucosa
Skin − Anterior nares
Oral − Supragingival plaque
Vaginal − Posterior fornix
P-value
10-11
10-8

Figure S9

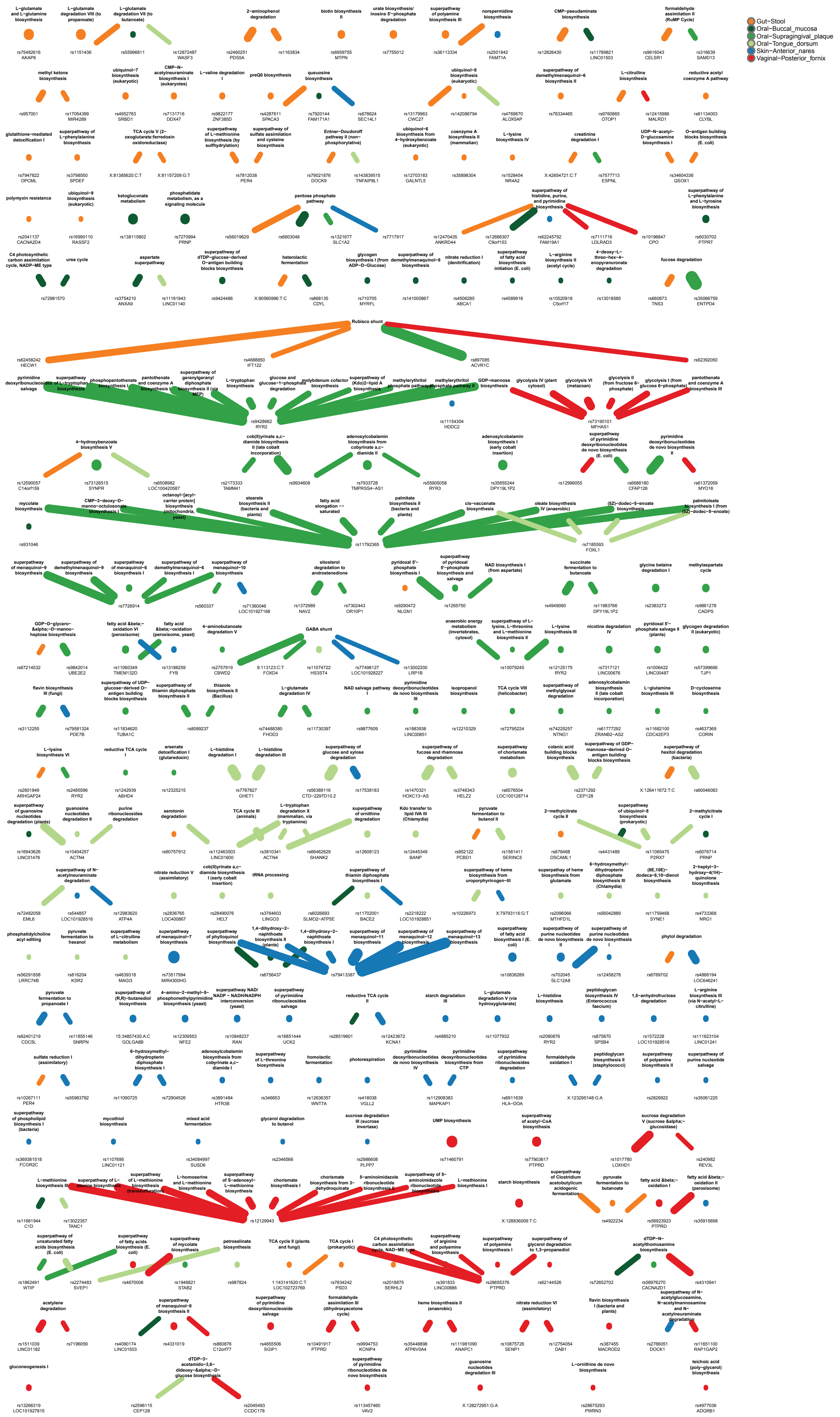
Gut−Stool
Oral−Buccal_mucosa
Oral−Supragingival_plaque
Oral−Tongue_dorsum
Skin−Anterior_nares
Vaginal−Posterior_fornix

MAF	Indel	Multi-allelic	SNV	Summary
AC1	974,836 (7.9%)	0 (0%)	11,375,702 (92.1%)	12,350,538 (41.7%)
AC2	258,462 (8.1%)	54,546 (1.7%)	2,870,739 (90.2%)	3,183,747 (10.7%)
MAF < 1%	267,166 (8.1%)	74,313 (2.2%)	2,968,917 (89.7%)	3,310,396 (11.2%)
1% < MAF < 5%	299,227 (8.0%)	125,840 (3.4%)	3,297,232 (88.6 %)	3,722,299 (12.6%)
MAF > 5%	547,164 (7.8%)	257,000 (3.6%)	6,250,382 (88.6%)	7,054,546 (23.6%)
**Summary**	**2,346,855 (7.9%)**	**511,699 (1.7%)**	**26,762,972 (90.3%)**	**29,621,526**

Table S1. Number of variants and minor allele frequency.

Variation	MAF	LoF	Moderate	Low
Indel	AC1	1,315	926	518
	AC2	228	185	130
	MAF < 1%	218	171	114
	1% < MAF < 5%	195	177	117
	MAF > 5%	299	243	233
	All	2,255	1,702	1,112
SNV	AC1	1,647	41,213	31,268
	AC2	236	7,981	7,462
	MAF < 1%	244	7,671	7,484
	1% < MAF < 5%	233	7,448	7,996
	MAF > 5%	310	9,863	13,326
	All	2,670	74,176	67,536

Table S2. Coding mutation distribution according to minor allele frequency and impact on gene product. LoF, loss of function.
